# Needs assessment for adapting TB directly observed treatment intervention programme in Limpopo Province, South Africa: A community-based participatory research approach

**DOI:** 10.4102/phcfm.v8i2.981

**Published:** 2016-07-22

**Authors:** Jabu T. Mabunda, Lunic B. Khoza, Hubertus B. Van den Borne, Rachel T. Lebese

**Affiliations:** 1 Department of Public Health, University of Venda, South Africa; 2 Department of Advanced Nursing Science, University of Venda, South Africa; 3 Health Education and Promotion, Maastricht University, the Netherlands

## Abstract

**Background:**

Limpopo Province is one of the hardest hit by tuberculosis and human immune virus infections in the country. The province has been implementing a directly observed treatment strategy since 1996. However, the cure rate was 64% in 2015 and remains far from the set target by the World Health Organization of 85%. Poor health-care seeking and adherence behaviours were identified as major risk behaviours.

**Aim:**

To apply a Community-Based Participatory Research (CBPR) approach in identifying barriers and facilitators to health-care seeking and adherence to treatment, and to determine strategies and messages in order to inform the design of an adapted intervention programme.

**Setting:**

This study was conducted in three districts in the Limpopo Province, Capricorn, Mopani and Sekhukhune districts.

**Methods:**

The community participatory research approach was applied. Purposive sampling was used to sample participants. Focus group discussions were used to collect data. Participatory analysis was used comparing findings within and across all the participants.

**Results:**

A total of 161 participated in the study. Participants included coordinators, professional nurses, supporters and patients. Major modifiable behavioural-related barriers were lack of knowledge about tuberculosis, misinformation and misperceptions cultural beliefs, stigma and refusal of treatment support. Environment-related barriers were attitudes of health workers, lack of support by family and community, lack of food and use of alcohol and drugs. Strategies and messages included persuasive and motivational messages to promote healthy behaviour.

**Conclusion:**

Joint programmatic collaboration between the community and academic researchers is really needed for interventions to address the needs of the community.

## Introduction

TB remains one of the public health challenges despite the fact that it is both preventable and treatable. TB ranks as the leading cause of death from infectious diseases worldwide after HIV. In 2014, ‘an estimated 9.6 million people developed TB and 1.5 million died from the disease: 1.1 million among HIV negative people and 390 000 of whom were HIV positive’.^1^ In the same year (2014), about 64% of the estimated 9.6 million people who developed TB were notified as newly diagnosed cases. About ‘3.6 million TB cases are said to have been left out either not diagnosed, or diagnosed but not reported to the National TB programme’. South Africa is ranked sixth among the 22 high-burden countries that constitutes 83% of the global incidence of TB cases with 410 000–520 000.^1^

Tuberculosis is a leading cause of death in Limpopo Province in 2015 among infectious diseases.^2^ Limpopo is one of the provinces hardest hit by TB and HIV in the country. Early diagnosis and treatment of TB-infected people is considered as a very effective control strategy. The basis of the World Health Organization’s Directly Observed Treatment Short-course Strategy (DOTS) is case finding and treatment by short-course chemotherapy under supervision.^3^ At its core, the strategy, Directly Observed Therapy (DOT) ensures that TB patients adhere to treatment.

However, many patients fail to adhere to treatment which leads to transmission of infection and development of multidrug resistance (MDR) TB. Reported MDR-TB cases increased from 8198 in 2008 to 14 161 in 2012. Extensive drug resistance (XDR) TB cases also increased from 488 cases in 2008 to 1545 in 2012.^4^ A much greater awareness about transmission, symptoms, prevention, health-seeking behaviour and adherence in Limpopo is required. Also, stigma was found to be associated with TB and HIV and has been cited in many studies as a predictor of negative health-seeking behaviour.^5,6,7,8,9^

A formative study was conducted to comprehensively identify determinants related to DOT implementation in Limpopo Province.^10^ The PRECEDE part of the PRECEDE PROCEED model^11,12^ guided the study in which the needs for development of the adapted TB DOT Programme Framework were identified. The study identified belated health-care seeking and non-adherence to TB treatment as major behavioural determinants of TB problems indicating the limitations of the present DOTS programme. In order to develop a culturally sensitive tuberculosis intervention (the adapted DOTS), a participatory study to elicit the perceived causes of non-health seeking and non-adherence to TB treatment was deemed necessary.

### Theoretical framework

**FIGURE 1:**
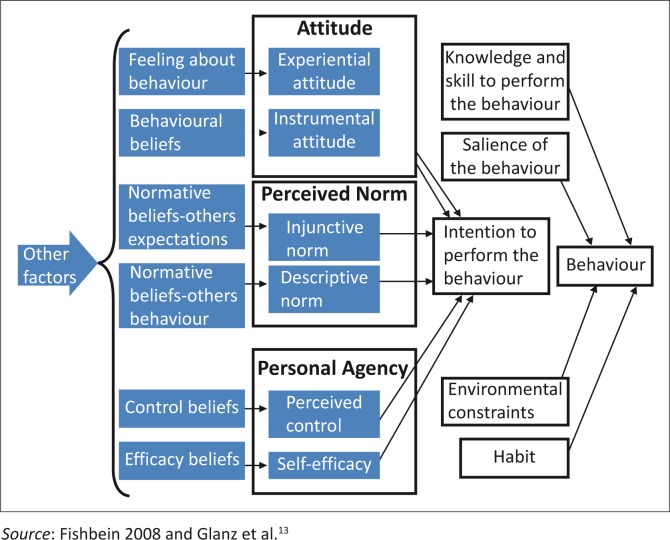
Integrated behavioural model.

An Integrated behavioural model (IBM)^13^ was chosen to guide this study. The theory includes constructs from the Theory of Reasoned Action and Theory of Planned Behaviour as well as other influential theories (Figure 1). The theory was chosen because it can be applied to diverse behaviours and population. The most important determinant of behaviour in the IBM is intention to perform a behaviour which follows reasonably from specific beliefs that people hold about the behaviour.^13^ Glanz et al. identified three components: (1) ‘a person has a strong intention to perform it and the knowledge and skill to do so, (2) there are no serious environmental constraint preventing performance, (3) the behaviour is salient, and (4) the person has performed the behaviour previously’.^13^ If a person has a strong intention to seek health-care, get tested and adhere to treatment, it is important to ensure that the person has knowledge on the signs and symptoms of the disease and the health-care system.

According to the model, behavioural intention is determined by three types of perceptions: *attitude, perceived norm* and *self-efficacy/perceived agency. Attitude*, if people are having positive attitudes related to TB, when they experience the signs of TB, they will more likely present for testing and adhere to treatment. *Perceived norm* is ‘a social pressure one expects from relevant others regarding performing the behaviour’. If friends and family members support or performing the behaviour themselves, the patient is likely to perform the behaviour also. *Self-efficacy* ‘is one’s perceived capability to successfully perform a specific behaviour’.^13,14^ If a TB patient has confidence and there is also control of the environment such as reduced stigma in the community, the person with symptoms is likely to seek care and adhere to treatment.

### Purpose of the study

The aim of this study was to apply a community-based participatory research approach in identifying barriers and facilitators to health-care seeking and adherence to treatment, and to determine strategies and messages in order to inform the design of an adapted DOT intervention programme. The objective was to explore barriers and facilitators to health seeking and adherence to treatment.

## Research methods and design

### Study design

A CBPR approach using qualitative methods was used to explore barriers and facilitators to belated care seeking and non-adherence to treatment behaviour in Limpopo Province. Workshops and focus group discussions were used in the study.

CBPR is a collaborative approach to participatory research. It is defined as ‘a collaborative process that equitably involves all partners in the research process and recognizes the unique strengths that each brings. The CBPR process begins with a research topic of importance to the community with the aim of combining knowledge and action for social change to improve community health and eliminate health disparities’.^15^ The aim of CBPR is to ‘increase knowledge and understanding of a given phenomenon and integrate the knowledge gained with interventions and policy change to improve the health and quality of life of community members’.^16^

The application of CBPR was perceived appropriate for this study because we expected its collaborative nature to enhance the implementation and adoption of the adapted DOTS programme. Equitable community participation helps to ensure that the programme focus reflects the concerns for the local community: it can bring a greater breath of skills, knowledge of community members, and community expertise to the project; and helps to make sure that the research topic reflects a major concern of the community, and will improve external validity of interventions.^12,17,18^

The CBPR has been implemented for the development of successful interventions before, for example in addressing social determinants,^19^ smoking cessation^20^ and explaining health disparities in diseases such as cancer, diabetes and cardiovascular.^21,22,23^ Researchers also used this approach with the purpose to enhance collaboration between research partners to identify needs and resources to empower communities.^24^

### Setting

The study was conducted in Limpopo, a rural province with five districts namely Capricorn, Mopani, Sekhukhune, Vhembe and Waterberg. Limpopo is the worst performing province in terms of TB outcomes. In 2013, the province reported a success rate of 57.6% and the cure rate of 64.0%. All the five districts performed badly in terms of success and cure rates. Only one district reached above 70.0% in both success and cure rates which are also far from the WHO target of 85.0% and the 90.0% of the BRICS countries. In terms of the defaulter rate only Capricorn reported above the set target (5.0%). Four of the districts are among the 10 high-death-rate districts in the country and above the 11.0% provincial death rate.^4^ Table 1 shows the performance in 2013 according to the districts.

**TABLE 1:** TB performance outcomes according to districts, Limpopo Province 2013.

District	Defaulter (%)	Success rate (%)	Cure rate (%)	Death rate (%)
Capricorn	4.4	50.2	69.5	12.4
Mopani	4.5	74.1	76.7	12.1
Sekhukhune	3.8	56.2	58.8	11.7
Vhembe	4.7	46.7	47.9	6.8
Waterberg	5.8	62.6	64.9	11.5
Limpopo	4.5	57.6	64.0	11.0
National	5.8	77.9	76.8	7.4

*Source*: Massyn et al.^4^

Public health institutions are the main providers of TB services, and the TB programme is integrated into the primary health-care services. The TB services are free and provided through fixed facilities and mobile teams. Community members can access free diagnostic and treatment services in public hospitals and clinics. Despite the accessibility and affordability of the TB services, people still present late and when put on treatment do not adhere.

### Population and sampling

A multistage sampling method was used. In the first stage, purposive sampling was used to sample three districts, namely Capricorn, Mopani and Sekhukhune, based on the knowledge that they had already been designated by the province as crisis districts, reporting cure rate of less than 50%. One sub-district from each district was then targeted based on the poor performance on treatment outcomes. The three sub-districts were Lepelle-Nkumpi in Capricorn, Greater Giyani in Mopani and Ephraim Mogale in Sekhukhune districts.

In the second stage, purposive sampling was used to sample participants for focus groups. TB coordinators, professional nurses and DOT supporters were invited to a meeting where the purpose of the study was explained and all were willing to participate therefore provided a written informed consent. The third stage involved convenient sampling of TB patients and community members. Patients and community members were invited to a meeting and asked to participate.

### Selection and recruitment of research participants

The first step was to identify a group that would constitute a planning team to work as partners with the researchers throughout the multiphase study. The planning team comprised of all TB coordinators in the province and included three provincial TB managers, five district TB coordinators and 25 sub-district TB coordinators. Their role included recruitment of participants and conducting focus groups. Those who agreed to participate provided written informed consent. The groups included professional nurses (37), DOT supporters (34), community members (30) and patients (27).

### Data collection

A workshop and focus groups were used to collect data. A workshop was conducted with the planning group. Focus group discussions were conducted with professional nurses, DOT supporters, community members and patients. Discussion agenda for the focus groups was developed through discussion with the planning team at a planning workshop prior to data collection.

### Workshop with the planning group

A highly participatory workshop was conducted to review the Adapted TB programme framework. The group comprised of 25 TB coordinators from the five districts in Limpopo Province. To prepare for the workshop, the researcher synthesised and organised a working document related to the determinants and performance objectives according to three components: health-care workers, DOT supporters and TB patients and community. Participants were also asked to review the policy and guidelines responsible for TB management in the province.

In order to accomplish the task, the group was to review each method and practical application, and then develop a programme component to deliver it. The group was divided into three according to the three components of the developed TB DOT Framework. The groups were facilitated by the provincial TB managers and the university team. Flip charts and power point presentations were used to document the discussions and presented to the whole group for further discussion.

Intervention mapping^12^ approach guided the generation of ideas about the type of programme intervention that can influence the desired programme objective. The idea behind the IM steps was to help in developing a programme that has a solid theory and evidence base. Planners are to find a balance between preliminary ideas on the one hand and theory and evidence-informed decisions on the other hand. This is very important as what the lay people think about the effectiveness of methods is not always congruent with available scientific evidence. Another reason is that evidence-informed methods and applications may not be easily applicable in the actual context of the programme or the potential participants.^12^

The first task for the planning team was to determine preference for programme design. Firstly, the researcher presented the two working styles to the group. According to Bartholomew et al.,^12^ there are two contrasting styles in this task, but neither of the styles means abandoning the previous work. The first style involves taking each method and its practical application, considering it and developing a programme component to deliver it. The second style is to put away all lists, matrices and other planning papers and allow the group to bring forth all ideas that group members have had to this point as to what the programme should look like.

An overview of the two styles was presented at the beginning of the workshop, and the groups were given the opportunity to choose the preferred style. The groups first referred to the methods, changed objectives and practical application to make sure the programme components are actually delivering what they are supposed to deliver. This style allowed creativity in determining programme components, how to implement them and what tasks will be required. All three groups chose the first style but later two groups changed to the second style. The reason for the change was that it was easy to explore ideas about the determinants, intervention messages, content and vehicle to deliver the messages.

### Focus group discussions: Professional nurses, community members, DOT supporters and TB patients

Focus groups were further used to identify and explore barriers and facilitating factors related to health-care seeking and adherence to TB treatment. During the focus groups, it was emphasised that the data gathering was to get a picture of the different groups so as to have a firm basis for realistic intervention planning. Twelve focus groups were conducted, three with each category (professional nurses, DOT supporters, patients and community members) from the three participating districts. The interview guide was designed as a thematic guide and development of the questions was the result of a thorough review of all the earlier collected materials focusing on attitudes, barriers and facilitating factors related to DOT, strategies and messages to promote care seeking and adherence to TB treatment behaviour.

Tsonga was used in the Greater Giyani municipality while Sepedi was used in Lepelle-Nkumpi and Sekhukhune municipalities. The focus groups lasted for about 2 h and were conducted in venues arranged by the TB coordinators. The focus groups were moderated by the research assistant, who was trained in focus group techniques and in participatory research methodology. Notes were taken by the researcher and the research assistant. Focus group discussions were audio taped and transcribed verbatim and translated. During the focus groups, data were summarised in flip charts, and presented to the rest of the groups for further comments and suggestions.

### Data analysis

Qualitative thematic analysis employing a participatory approach was used and included comparing findings within and across the three sub-districts categories. Data were manually compiled and tabulated by the researcher into a matrix according to the themes. Notes compiled during focus groups were used to verify the data. Data were read repeatedly to identify patterns and draw conclusions. The analysis summaries were validated through a member-checking process in which the reports were reviewed with the planning team to determine accuracy of interpretations.

### Ethical consideration

Ethical clearance was granted by the Ethics Committee of the University of Venda and the permission to conduct the study sought from the Provincial Department of Health. The participants voluntarily gave a verbal informed consent as an agreement to participate in the study. The researcher ensured respect and protection to research participants through assurance of confidentiality of information shared. Although confidentiality and anonymity in participatory research is quite difficult to achieve, the researcher established trust and mutual respect within the research participants so that information can be shared without risk of harm to those concerned. Participants in focus groups were provided with refreshment and those who used and paid transport were reimbursed for their transport as per tickets cost.

### Trustworthiness

Lennie^25^ proposes that increasing rigour and trustworthiness of participatory research clearly requires the use of methods, criteria and strategies that are appropriate to skills. TB managers and coordinators participated in the study so they provided particularly useful assistance in identifying all relevant key people. A number of strategies were used to increase rigour and trustworthiness. Building mutual trust and open communication through actively listening to participants in an empathetic way, facilitating discussions and gathering continuous feedback on the research. The partners managed to build rapport with each other. The participants were involved in data analysis to ensure credibility.

## Results

### Characteristics of the participants

A total of 161 participated in the study, 33 participated in a workshop and 128 participated in focus group discussions. The participants in the workshop comprised of 3 provincial, 5 district and 25 sub-district TB coordinators. Table 2 provides the demographic characteristics of participants in focus groups. Twelve focus groups were conducted with 37 professional nurses, 37 DOT supporters, 34 DOT supporters, 30 community members and 27 patients. Females constituted 108 of the participants and age ranged from 19 to 59 years. Only 28 participants among the patients and community members had primary and lower level of education, while the majority had secondary and above.

**TABLE 2:** Demographic characteristics of participants from the three local areas combined.

Variable	Prof nurses (*n* = 37) *n* (%)	DOT supporters (*n* = 34) *n* (%)	Community members (*n* = 30) *n* (%)	Patients (*n* = 27) *n* (%)
**Gender**				
Female	35 (94.6)	30 (88.2)	24 (8.0)	19 (70.3)
Male	2 (5.4)	4 (11.8)	6 (20.0)	8 (29.6)
**Age**				
19–29	2 (5.4)	3 (8.8)	2 (6.7)	6 (22.2)
30–39	28 (75.7)	20 (58.8)	15 (50.0)	12 (44.4)
40–49	6 (16.2)	9 (26.5)	11 (36.7)	9 (33.3)
50–59	1 (2.7)	2 (5.9)	0	1 (3.7)
60 +	-	-	2 (6.7)	-
**Highest qualification**				
Abet	-	-	2 (6.7)	4 (14.8)
Primary	-	-	8 (26.7)	14 (51.9)
Secondary	-	3 (8.8)	10 (33.3)	8 (29.6)
Matric	-	30 (88.2)	6 (20.0)	1 (3.7)
Tertiary	37 (100.0)	1 (2.9)	4 (13.4)	-

DOT, Directly Observed Treatment.

The results from all the participants are presented together as they all focused on the three components of the framework (community, health-care workers, and patients and DOT supporters). The results are presented under the two major themes, health-seeking behaviour and adherence to treatment. The themes are further divided into three sub-themes: barriers, facilitation strategies and messages (Table 3).

**TABLE 3:** Summary of the findings of the focus group discussions.

Theme	Barriers	Facilitation strategies/materials	Messages
Health-seeking behaviour	Lack of knowledge. Cultural beliefs. Stigma (discrimination). Consulting traditional and private practitioners. Not having cough.	Door-to-door campaign. Health talks (schools, shebeens, etc.). Imbizo. Local media like radio, TV, communication materials, DVDs/video and billboards.	TB can happen to anybody. Together we can beat TB. If having symptoms get tested for TB. Only TB medications kills TB germ.
Non-adherence to treatment	Lack of knowledge. Cultural beliefs. Stigma. Mixing traditional herbs and TB medication. Smoking and alcohol use. Refusing DOT. Side effects. Denial. Social grant.	Initial and on-going counselling. Use TB ambassador. Life style modification. Home visit. Patient select supporter. Support groups. Fast queue. Accept and disclose status. Family support.	TB disease (what it is, symptoms, treatment). Importance of having DOT supporters. TB can be cured. Only TB medication kills TB germ. Think about your life.

DOT, Directly Observed Treatment.

### Perceived barriers to health-seeking behaviours

The barriers related to the community included attitudes of community members towards the clinic or staff members, lack of information about TB, cultural beliefs such as witchcraft and associating TB with uncleanliness resulting from sleeping with a woman following an abortion (culturally known as ‘makgoma’). These factors contributed to people who are TB-infected consulting private and traditional practitioners first. Sometimes the private health practitioners fail to diagnose TB.

One participant reported:

‘I don’t know, people still believe in witchcraft … they think they are bewitched and consult traditional healers … those with medical aid consult private doctors … they don’t know is TB.’ (Supporter 10)

Participants reported that self-medication prior to seeking medical help was widespread among community members. For example, using traditional medicines such as ‘moringa’ or other immune boosters bought from pharmacies especially by those with medical aid. Prayer was also raised as another factor delaying community members from seeking medical help. These factors have been reported as a strong predictor of prolonged delay in seeking care as well as defaulting treatment.

One participant reported:

‘Nowadays people believe in prayer … they think with prayer they will be cured … they take immune boosters like ’moringa’ … only when they are very sick that they are forced to come to the hospital.’ (Supporter 6)

Social stigma was also reported as a barrier. People fear that if they seek medical help may be told that they have HIV and/or AIDS.

One participant said:

‘is not easy … people say that if you have TB, you test for HIV … so I think that make people not to go to hospital.’ (Community member 22)

### Potential barriers for defaulting treatment

Cultural beliefs again played a big role in adherence to TB medications. Among the factors raised were that traditional practitioners claim that they can cure TB and stop patients from taking treatment. Factors related to TB patients included lack of knowledge about the disease, lack of food, treatment and side effects. Patients reported that on discharge from the hospital they are not told why treatment took so long and the reason for a DOT supporter. These factors negatively affected adherence to treatment because they even failed to report side effects because they were never told about them and what to do if they experience the side effects.

One participant reported:

‘when I feel better or not feeling better when taking the drugs … I stop … these drugs sometimes make a person feel tired, eat a lot … where will you get food to eat every now and then … cannot take drugs on an empty stomach?’ (Patient 16)

Environmental-related factors reported included the attitudes of health staff members, lack of DOT support mapping where the clinics do not have a list of supporters, stigma, and lack of family and community support. Due to stigma attached to TB, most patients refuse treatment supervision, giving wrong addresses or hiding in the house not opening the door for treatment support.

One participant reported:

‘we are not allocated TB patients … some clinics do not have our pictures … is like clinic staff does not trust us.’ (Supporter 12)

One participant reported:

‘I don’t trust home-based carers … they gossip about people’s sicknesses … you can’t trust them.’ (Patient 23)

Another participant said:

‘hey, some do not see the need for a carer … frequent visits by a carer is almost disclosing your status.’ (Patient 16)

Other factors raised in one DOT supporters’ focus group were related to gender where male patients would prefer to have a male DOT supporter. Socio-economic factors included lack of food, smoking and alcohol use, lack of knowledge about TB and treatment, lack of confidentiality, time spent waiting for medication and long queues.

### Suggested strategies and messages

Facilitation strategies and messages to encourage community members included door-to-door campaigns to improve knowledge regarding TB, transmission, prevention and adherence to treatment. Community health promotion activities to improve care seeking and adherence to treatment should include the media (radio, print and television) and health education in schools, churches and other community-gathering places. To improve disclosure, TB patients should join support groups, continuous counselling is also recommended for TB patients. Other strategies included use of TB ambassadors (those that are cured of TB) to educate and identify TB suspects, and providing stipend to DOT supporters. The idea of DVDs to be used for education was highly endorsed by participants across the groups.

Messages suggested to promote timely health-seeking behaviour and adherence to treatment included, for example, TB can happen to anybody, TB is curable, take your treatment and be cured, avoid alcohol and drugs, disclose your TB status, and immune boosters cannot kill the TB germ.

## Discussion

The findings of the study confirm those from the previous study within the SANPAD project.^10^ The findings revealed that TB control in Limpopo is constrained by varied barriers. Limited knowledge about TB results in delays in seeking care and increase the risk of TB transmission. In a cluster randomised control trial study conducted in KwaZulu-Natal, the results showed a strong association between knowledge of signs and symptoms, transmission and prevention, and positive health-seeking behavioural intentions for the patients themselves and the family members.^26^

Treatment interruption is also related to perceptions about TB as a disease. Perceptions about the disease influence seeking behaviour. Understanding how people interpret TB causes and its symptoms help health-care providers understand why people may delay seeking treatment. Several studies showed that, associating TB symptoms with HIV or AIDS, delayed seeking treatment for fear of being told that they are HIV positive.^27,28,29^

TB is a complex health problem and therefore demands a multilevel components intervention. The intervention components need to target the behaviour of TB patients and the environment (community members and health-care workers) focusing on motivation to change the negative attitude toward TB. The magnitude of the challenges justifies the adoption of participatory approach involving various stakeholders to develop an intervention to meet the community needs. Participatory design of the recommended strategies and materials may correct misinformation about TB among community members, misconceptions about TB transmission and care, reduce stigma and increase trust of health services.

## Conclusion

The study provided needs and suggestions on risk behaviours that undermine TB control efforts in Limpopo. The magnitude of the challenges justifies the adoption of participatory approach involving various stakeholders to develop an intervention to meet the community needs. Joint programmatic collaboration between the community and academic researchers is really needed. Participation may also help to instill a sense of ownership and also ensures long term sustainability and adoption of the intervention.
